# Enhanced Photocatalytic Activity of {110}-Faceted TiO_2_ Rutile Nanorods in the Photodegradation of Hazardous Pharmaceuticals

**DOI:** 10.3390/nano8050276

**Published:** 2018-04-25

**Authors:** Tran Thi Thuong Huyen, Tran Thi Kim Chi, Nguyen Duc Dung, Hendrik Kosslick, Nguyen Quang Liem

**Affiliations:** 1 Institute of Materials Science (IMS), Vietnam Academy of Science and Technology (VAST), 18 Hoang Quoc Viet, Cau Giay, Hanoi 100000, Vietnam; chittk@ims.vast.ac.vn (T.T.K.C.); liemnq@ims.vast.ac.vn (N.Q.L.); 2 Institute of Chemistry, Department of Inorganic Chemistry, University of Rostock, Albert-Einstein-Str. 3a, Rostock 18051, Germany; hendrik.kosslick@uni-rostock.de; 3 Advanced Institute of Science and Technology (AIST), Hanoi University of Science and Technology, 1 Dai Co Viet, Hanoi 100000, Vietnam; dung.nguyenduc@hust.edu.vn

**Keywords:** rutile, active facets, pharmaceutical, degradation, mineralization, active species

## Abstract

Rutile TiO_2_ with highly active facets has attracted much attention owing to its enhanced activity during the photocatalytic degradation of pollutants such as pharmaceuticals in wastewater. However, it is difficult to obtain by controlling the synthetic conditions. This paper reports a simple hydrothermal synthesis of rutile TiO_2_ nanorods with highly exposed {110} facets. The obtained rutile was characterized by X-ray diffraction (XRD), scanning electron microscopy (SEM), high-resolution transmission electron microscopy (HR-TEM), and Raman spectroscopy. The main contribution to the photocatalytic activity comes from rutile nanorods with highly dominant active {110} facets, which were studied in the photodegradation of reactive cinnamic acid and more recalcitrant ibuprofen. The contribution of active species was also investigated. The present work further confirmed the hydrothermal synthesis route for controlling the preparation of highly crystalline and active rutile nanocrystals.

## 1. Introduction

Heterogeneous photocatalysis on semiconductor photocatalysts has attracted considerable interest due to its applicability in the treatment of hazardous organic pollutants [1,2,3,4,5]. Among the ultraviolet light driven photocatalysts, TiO_2_ has received much attention. The use of a TiO_2_ photocatalyst and a wide band gap (~3.2 eV) [6] offers several advantages including its low cost, chemical stability, high oxidizing ability, safety, and reusability [7,8,9]. TiO_2_ occurs in three main phases: anatase, brookite, and rutile, with anatase being the most commonly used in photocatalytic applications [10,11,12,13,14]. Recently, some photocatalytic studies on rutile TiO_2_ have been published. Kalaivani’s group reported the enhanced photocatalytic decolourization efficiency of methylene blue over the bio-nanocomposite inulin-TiO_2_ rutile under ultraviolet (UV) irradiation [15]. In this case, the inulin-TiO_2_ rutile was obtained by embedding rutile nanoparticles into a novel biopolymer-inulin. The approach allowed the diminishing of the agglomeration of rutile nanoparticles, providing a larger surface area, thus improving the activity of rutile. In another report, Nair’s group found that the high photocatalytic activity of three-dimensional rutile micro-flowers in the decolourization of Rhodamine B under UV light was due to the large surface area contributed by the highly dense spiky nanostructures [16]. In terms of enhanced activity, rutile nanocrystals with exposed active facets are relatively unknown due to its difficulty in synthesis. Therefore, acquiring a large percentage of active facets by controlling the synthetic conditions is highly desirable. According to the nature of rutile rods, the crystal growth is indeed a result of the competitive growth of {111} facets and {110} facets [17,18]. The reported rutile with exposed {111} facets that had both the advantages of large specific surface area and exposed high active facets were active in the decolourization of methylene blue under UV irradiation [19]. In our work, the large-sized rutile nanocrystals with highly exposed {110} facets were successfully prepared by hydrothermal synthesis and exhibited an enhanced activity in the photodegradation of reactive cinnamic acid (CA) and recalcitrant pharmaceutical ibuprofen (IBP). The photocatalytic performances were investigated under low power solarium lamps, high organic loading, and a small amount of the photocatalyst. Such testing conditions have been rarely reported in wastewater treatment. In addition, trapping experiments were carried out to analyze the contribution of active species to the photodegradation of organic compounds.

## 2. Materials and Methods

### 2.1. Materials

All chemicals were of analytical grade and used without further purification: titanium (IV) *i*-propoxide (TTIP, Merck, Kenilworth, NJ, USA, 98%), hydrochloric acid (HCl, Chemsolute, Th. Geyer, Berlin, Germany, 35–38%), cinnamic acid (C_9_H_8_O_2_, Reachim, Moscow, Russia, 99%), ibuprofen sodium salt (C_13_H_17_O_2_Na, Sigma-Aldrich, St. Louis, MA, USA, 98%), ethylenediaminetetraacetic acid (C_10_H_16_N_2_O_8_, Sigma-Aldrich, 99%), *i*-propanol (C_3_H_8_O, Sigma-Aldrich, >99%), tert-butanol (C_4_H_10_O, Sigma-Aldrich, 99%), benzoquinone (C_6_H_4_O_2_, Sigma-Aldrich, ≥98%), and titania P25 (TiO_2_, Evonik, Essen, Germany, 99.5%).

### 2.2. Synthesis of TiO_2_ Rutile

The rutile was prepared based on a previously reported procedure in [20] with improved synthesis parameters. The procedure consisted of two main steps:

(i) *Sol-gel synthesis of an amorphous titania precursor*: 20 mL of titanium (IV) *i*-propoxide was dissolved in 105 mL of *i*-propanol. The solution was kept at 0 °C under vigorous stirring. To this colorless solution, a stock solution containing 105 mL of *i*-propanol and 1 mL of distilled water prepared at room temperature (RT) was slowly dropped over a period of 5 h. The suspension gradually changed into a white/milky color. This was further stirred at RT for 24 h. Once the reaction was completed, the white product was removed from the suspension by centrifugation and the obtained clear colorless solution was again diluted with 1000 mL of distilled water and further stirred at RT for 24 h. The obtained white amorphous titania was separated by centrifugation and washed with distilled water and ethanol, then dried under vacuum at 60 °C. The final white powder was used as the precursor for the hydrothermal treatment step.
(ii) *Hydrothermal treatment of amorphous titania precursor*: 1.0 g of amorphous titania was placed into a 120 mL Teflon cup and then an appropriate amount of concentrated aqueous 4.0 M hydrochloric acid was added and stirred at RT for 30 min. Next, the Teflon cup was transferred into a stainless steel-lined autoclave, which was placed into an oven and heated at 200 °C for 7 h. Thereafter, the autoclave was allowed to cool down to RT. The precipitate was decanted from the reaction mixture, washed thoroughly with distilled water and ethanol, and finally dried at 60 °C overnight in an oven. The final product was ground in a porcelain mortar with a pistil to obtain fine powders.

### 2.3. Characterization

The morphology and microscopic structure of the samples were characterized by scanning electron microscopy (SEM) (FE-SEM S-4800, Hitachi, Tokyo, Japan) operating at 5 kV, and transmission electron microscopy (TEM) and high-resolution transmission electron microscopy (HR-TEM) (JEM 2100, JEOL, Tokyo, Japan) operating at 200 kV.

The crystal structures and phases of the samples were measured using an X-ray diffractometer (STADI-P, STOE, Darmstadt, Germany) with monochromatic Cu Kα radiation (*λ* = 1.5406 Å). Raman spectra were recorded using a LabRAM HR 800 Raman microscope system (Horiba Jobin YVON, Kyoto, Japan) equipped with a high stability BX40 microscope (Focus 1 μm). A blue laser (473 nm, 20 mW air-cooled solid-state laser) was used as an excitation source.

Brunauer-Emmett-Teller (BET) surface areas (S_BET_) were determined using the adsorption data in the relative pressure (*p*/*p*_0_) range of 0.05–0.35. The measurements were performed at 77 K on a Thermo Sorptomatic 1990 nitrogen adsorption apparatus (Thermo Fisher Scientific, Waltham, MA, USA).

The weight loss of the samples was evaluated from the thermogravimetric curve analyzed on a TGA Labsys 1600 DSC instrument (Setaram, Caluire, France) under argon gas at a heating rate of 10 K/min where 0.1 cm^3^ alumina (Al_2_O_3_) crucibles were used.

### 2.4. Photocatalysis

The photocatalytic performance of the rutile samples was evaluated in the photocatalytic degradation reactions of CA and IBP under ultraviolet-visible (UV-Vis) irradiation using batch-conditions. In each experiment, a glass beaker containing 10 mg of the photocatalyst and 250 mL of an aqueous 10 ppm organic solution was used. The reaction mixture was magnetically stirred in the dark at RT for 30 min to reach the adsorption-desorption equilibrium. Four UV-Vis solarium lamps with a total power of 60 W were used as the light source. These lamps simulate the UV part of sunlight (by light energy distribution and intensity) and emit a continuous spectrum range of about 370–400 nm. The distance between the applied lamps and the surface of the pollutant solution was 15 cm. Parallel tests were performed by placing four batches into a closed aluminum box. Four magnetic stirrers were arranged below. After certain time intervals (0 min, 15 min, 30 min, 1 h, 2 h, 3 h, 4 h, and 5 h), 5 mL aliquots were taken from the reaction mixture, and 5 mL aliquots were taken from the reaction mixture with a syringe and separated from the catalyst by a 0.45 μm polytetrafluoroethylene (PTFE) syringe filter. The abatement of IBP and CA was determined by the change in the absorbance at 221 and 273 nm, respectively, as follows [21]:

abatement (%) = (*A_0_* − *A_t_*)/*A_0_* × 100, (1)

where *A_0_* and *A_t_* are the initial absorbance and the absorbance after various time intervals of UV-Vis irradiation (*t*), respectively. All data were measured at RT using a Lambda 19 UV/Vis spectrometer (Perkin Elmer, Waltham, MA, USA).

The trapping experiments using different scavengers (1.46 mg of ethylenediaminetetraacetic acid (EDTA) as scavenger for holes, 0.1 mL of tert-butanol (*t*-BuOH) as scavenger for hydroxyl radicals, and 2.7 mg of benzoquinone (BQ) as scavenger for superoxide anion radicals) were performed in a similar manner to the above photocatalytic degradation reaction of IBP and CA except that the mentioned scavengers were added to the reaction.

## 3. Results

### 3.1. Characterization

**Figure 1:**
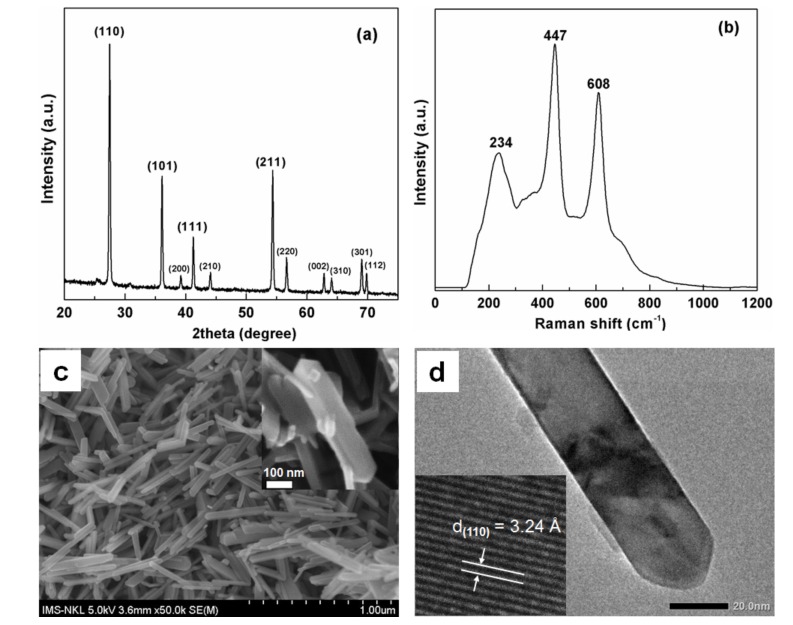
(**a**) XRD pattern; (**b**) Raman spectrum; and (**c**) SEM image of the as-synthesized rutile nanorods. The inset of (**c**) is the corresponding high-resolution SEM image; (**d**) Low resolution TEM image of rutile with exposed {110} facets. Inset: HR-TEM image taken from the body of the rutile nanorods.

A combined study of the XRD pattern and Raman scattering spectrum was performed to confirm the formation of the pure rutile phase. The XRD pattern (Figure 1a) revealed that the obtained hydrothermal product was present in the rutile form with a high intense diffraction peak at 27.4° (2θ) corresponding to the {110} facets and others with high intensities located at 36.1°, 41.2°, and 54.3° representing the {101}, {111}, and {211} facets, respectively [16,19]. The results were consistent with the theoretical diffraction pattern from the JCPDS database (No. 96-900-7532). The average crystallite size D_XRD_ of the rutile was calculated using the Scherrer equation from the width of the most intense rutile reflection (2θ = 27.4°) [22] and determined to be about 90 nm. Representative Raman spectrum (Figure 1b) showed that three bands appeared at 234, 447, and 608 cm^−1^, which are characteristic of the TiO_2_ rutile phase [23,24]. 

The SEM image of the rutile nanocrystals is shown in Figure 1c. Under the present hydrothermal treatment conditions, nearly uniformed rutile nanorods were formed with the size of about 50–100 nm in width and about 300–500 nm in length. Each nanorod consisted of four lateral smooth facets and two pyramidal ends (inset of Figure 1c), which were confirmed by the TEM image shown in Figure 1d. The inset of Figure 1d revealed that the main exposed facets of the obtained rutile were {110} facets corresponding to a spacing value of 3.24 Å.

Based on the weight loss and BET surface [25], the densities of the adsorbed water (physisorbed water) and surface hydroxyl groups (chemisorbed OH groups) were calculated in the temperature range of RT–250 °C and 250–700 °C, respectively [26]. The amount of adsorbed water was estimated as follows: (weight loss × Avogadro’s number)/(molecular mass of water × S_BET_), while surface hydroxyl groups were calculated as follows: (weight loss × Avogadro’s number)/(molecular mass of OH group × S_BET_). The rutile was covered with about twice the amount of OH groups than with anatase and titania P25 (Table 1). This property correlated with the surface hydroxylation, which might explain the difference in the photocatalytic performances between rutile and anatase [27].

### 3.2. Photocatalysis

#### Photocatalyic Activity

**Figure 2:**
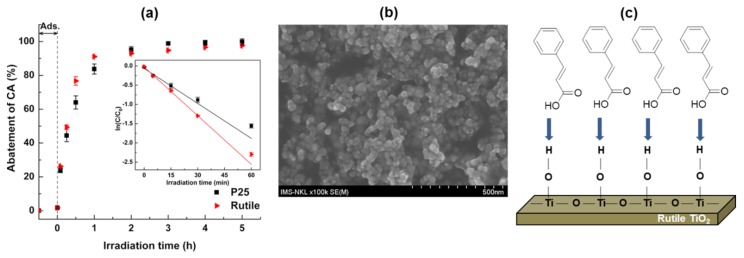
(**a**) Photocatalytic abatement of cinnamic acid (CA) in the photocatalytic performances of TiO_2_ P25 and rutile, inset: corresponding plots for linear fitting followed by the apparent first-order reaction model. Reaction conditions: RT, 10 ppm CA, 250 mL reaction solution, 10 mg catalyst loading; (**b**) SEM image of TiO_2_ P25; (**c**) Langmuir-Blodgett assembly of CA molecules on the planar crystal rutile facets.

The photocatalytic performance of rutile was investigated in the degradation of CA. For comparison, an identical assay was conducted using a commercial TiO_2_ (P25, Evonik, Essen, Germany). Figure 2a shows the CA abatement curves determined by the change in the CA absorbance. The CA adsorption on the surfaces of the rutile and P25 was similar (about 1.7%). Through a comparison of the specific surface area of rutile (12 m^2^/g) and TiO_2_ P25 (46 m^2^/g) determined by the BET method using the relative pressure range of 0.05–0.35 in the present nitrogen adsorption-desorption measurement (Table 1), the loading of CA molecules on the rutile surface was higher than that on the P25 surface. Obviously, the quite large planar facets of the rutile crystals allow for the alignment of an increased amount of CA molecules adsorbed on its surface. Such high loadings can be realized with aligned adsorbed molecule multilayers (Langmuir Blodgett-type) [28]. Therefore, the photocatalytic degradation of CA over low surface area rutile is not limited by mass transfer or adsorption.

After the first hour of exposure to UV irradiation, the rutile had slightly higher photocatalytic activity than the P25 (about 91% and 84% of CA was degraded with rutile and P25, respectively). Based on the Langmuir-Hinshelwood first-order kinetic model, the apparent first-order rate constant was derived from the slope of the linear transform ln(*C*/*C_0_*) = *f*(*t*), where *C_0_* is the initial concentration of CA and *C_t_* is the concentration of CA at various irradiation times (0 min, 5 min, 15 min, 30 min, and 60 min). Rutile exhibited a fast reaction rate of 0.04214, which was higher than that of the P25 (i.e., 0.03027) as seen in the insert of Figure 2a. A similar high CA abatement (98–99%) was achieved at the end of the reaction (duration 5 h) showing the unexpectedly high photocatalytic activity of the as-synthesized rutile even with its large crystal size and low surface area. It should be noted that the TiO_2_ P25 photocatalyst, being a mixture of anatase (80%) and rutile (20%), consisted of spherical aggregated nanoparticles with a diameter of about 25 nm (Figure 2b). In a reported comparison with spherical anatase TiO_2_ nanoparticles prepared by a similar hydrothermal procedure [26], rutile was more active for the CA photodegradation. Anatase had a much smaller particle size (10 nm) and higher BET surface area (132 m^2^/g). These findings showed that the shape, particle size, and surface area were not limiting factors when explaining the high activity of rutile in the CA photodegradation.

**Figure 3:**
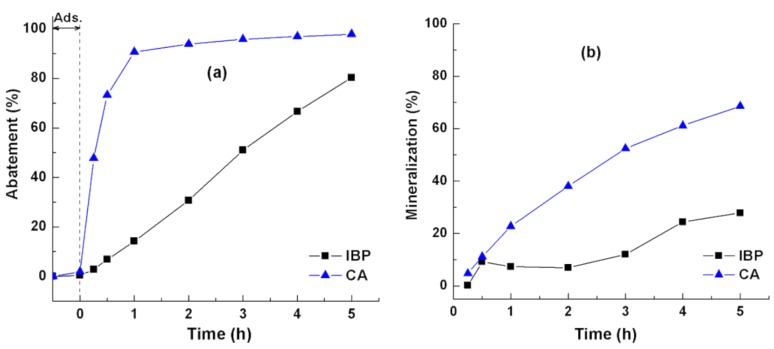
(**a**) Abatement and (**b**) mineralization of (CA) cinnamic acid and (IBP) ibuprofen in the photocatalytic performance of rutile. Reaction conditions: RT, 10 ppm pollutant, 250 mL reaction solution, 10 mg rutile loading.

To further understand the photocatalytic behavior of rutile, the degradation of IBP, known as a recalcitrant compound [29], was studied. Figure 3 shows the abatement and mineralization curves determined by the change in the absorbance of CA, IBP, and in the total organic carbon (TOC) removal, respectively. Under UV irradiation, the CA and IBP molecules were degraded immediately, especially with cinnamic acid (Figure 3a). In the photodegradation of IBP, the aromatic ring opening occurred gradually with irradiation time. After 5 h, about 80% of IBP and 98% of CA were degraded. The obtained abatement showed that rutile exhibited different photocatalytic activities depending on the reactivity or recalcitrance of the organic compounds. Namely, the reactive olefinic (–C=C–) double bond presented in the side chain of the CA molecules was immediately attacked at the onset of photocatalytic reaction [30]. As a result, the CA was nearly completely degraded after 5 h of reaction, while the abatement of IBP might take a longer time. This behavior was more pronounced in the mineralization. Figure 3b shows the markedly lower degree of mineralization when compared to the rapid degradation rate. This might be due to the formation of reaction intermediates and/or by-products such as hydroxylation products, ring-opened products, etc. About 98% (UV) and 69% (TOC) were achieved in the CA degradation after 5 h of the photocatalytic reaction, whereas about 80% (UV) and 28% (TOC) were obtained in the IBP degradation. These results indicated that rutile behaves according to the chemical reactivity of the organic compounds. In this work, the recalcitrance of the studied organic compounds increased as follows: CA < IBP.

The difference in the activity of rutile for the photodegradation of CA and IBP can be clarified in terms of the contribution of active species which are considered as practically involved in the photocatalytic reactions but have not been reported so far [31,32,33,34]. The role of active species was investigated by the impact of adding different scavengers (*t*-BuOH for ^•^OH scavenger, EDTA for holes scavenger, and BQ for $O_{2}^{●-}$ scavenger) on the photodegradation of IBP over rutile. 

**Figure 4:**
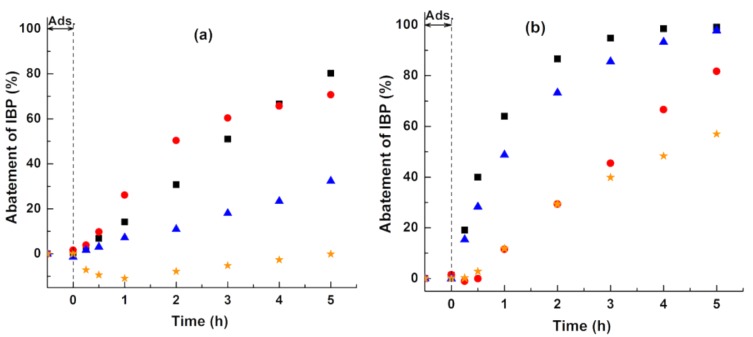
Impact of different scavengers on the photocatalytic abatement of IBP over (**a**) rutile and (**b**) anatase. Reaction conditions: RT, 10 ppm IBP, 250 mL aqueous reaction solution, 10 mg catalyst loading. No scavenger: (black square). Scavengers: (red circle) EDTA, (blue triangle) *t*-BuOH, and (orange star) BQ.

Figure 4a shows that the addition of *t*-BuOH remarkably reduced the photocatalytic IBP abatement from the original 80% to 30%, indicating the strong contribution of ^•^OH radicals in the treatment with rutile. In contrast, the addition of a holes (h^+^) scavenger had an unexpectedly increase in the abatement of IBP, e.g., from 30% (without addition of EDTA) to 50% (addition of EDTA) after 2 h of reaction. This means that these holes were not directly involved in the oxidation of IBP and can migrate to the rutile surface and react with surface OH groups and/or water molecules surrounding the rutile particles that ultimately lead to the formation of ^•^OH radicals [5,34,35]. The increase in the abatement of IBP after the addition of holes also implied that EDTA injected more electrons into the valence band of rutile, thereby improving charge carrier separation by the excitation of electrons to the conduction band, thus causing an enhancement in the formation of $O_{2}^{●-}$ radical anions via the reduction of molecular oxygen by electrons. As a result, the photodegradation of IBP was completely inhibited after the addition of the $O_{2}^{●-}$ scavenger.

In contrast with the photocatalytic performance of rutile, the holes contributed remarkably to the cleavage of the aromatic ring for the photodegradation of IBP treated with anatase, especially at the initial stage of reaction (Figure 4b). Obviously, the impact of ^•^OH radicals was minor. These results indicated that the lower activity of rutile when compared to anatase in the experiment without the addition of scavengers was due to the absence of oxidative holes. Interestingly, the $O_{2}^{●-}$ anions had a strong impact during the course of treatment with both photocatalysts (rutile and anatase).

By means of the scavenger experiments, we found that the holes were not directly involved in the photodegradation of IBP over rutile, but that they produced a relatively high number of ^•^OH radicals that acted as “door openers” via the oxidation of surface OH groups which were calculated and shown in Table 1. Murai et al. also suggested that holes were trapped at the titania surface in the adsorbed OH groups yielding the reactive ^•^OH radicals [36]. These findings explained the lower activity of rutile for the photodegradation of recalcitrant ibuprofen when compared to that of reactive cinnamic acid. Cinnamic acid does not require strong active species like holes but reacts with ^•^OH radicals to attack its olefinic double bond, therefore yielding a higher photodegradation efficiency.

**Figure 5:**
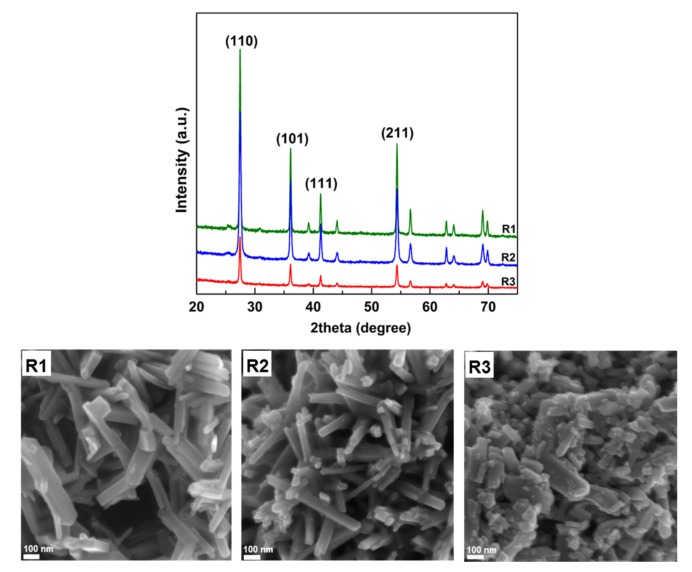
(**Top**) XRD and (**bottom**) SEM images of different rutile samples. R1, R2, and R3 represent the rutile synthesized by the hydrothermal method, rutile separated from the brookite/rutile mixture by peptization with water, and the rutile obtained by short-time grinding of R1, respectively.

In terms of the contribution of the active facets, the difference in the activity of rutile was further studied. Three different rutile samples were used for this purpose including R1 (directly synthesized by the hydrothermal method), R2 (separated from the brookite/rutile mixture by peptization with water), and R3 (obtained by the short-time grinding of R1). The morphology, crystal structures, and phases of the rutile samples are shown in Figure 5. Their XRD patterns indicated that the pure rutile phase was formed and well crystallized. Using the SEM data, the order of particle size was as follows: R1 > R2 > R3.

**Figure 6:**
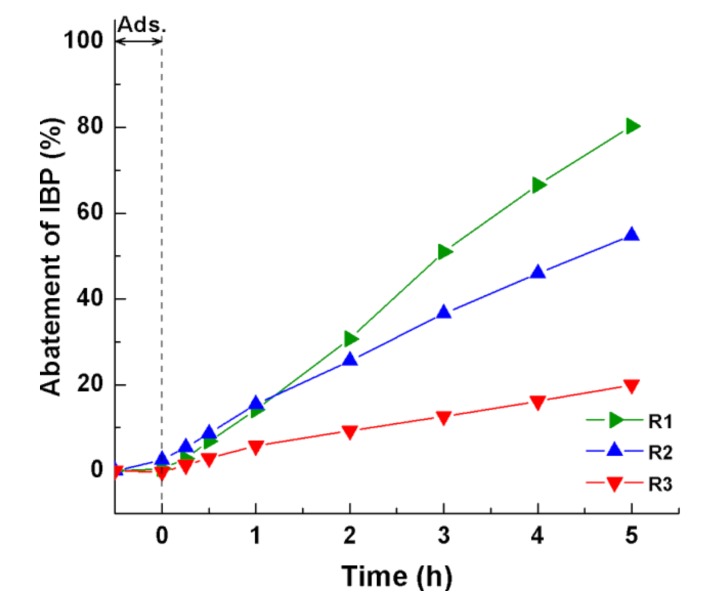
Photocatalytic abatement of IBP over different rutile samples (R1, R2, and R3 represent the rutile synthesized by the hydrothermal method, rutile separated from the brookite/rutile mixture by peptization with water, and the rutile obtained by the short-time grinding of R1, respectively). Reaction conditions: RT, 10 ppm IBP, 250 mL aqueous reaction solution, 10 mg rutile loading.

Figure 6 shows the photocatalytic abatement of IBP over different rutile samples. The difference in the activity between these samples was clearly observed. Only about 20% of IBP over R3 was degraded after 5 h under UV irradiation, while a 2–4 times higher IBP abatement was achieved with R2 (55%) and R1 (80%), respectively. A decrease in the photocatalytic activity followed in the order: R1 > R2 > R3, which was not dependent on the order of particle size: R1 > R2 > R3. The largest rutile nanorods (R1) with the highest activity indicated the minor role of particle size. The photocatalytic activity of rutile nanorods was clearly independent of particle size for the photodegradation of cinnamic acid (Table 2). In general, the particle size reduction accompanied by an increase in the specific surface area led to an enhancement in the activity of the photocatalysts [35,37].

**Figure 7:**
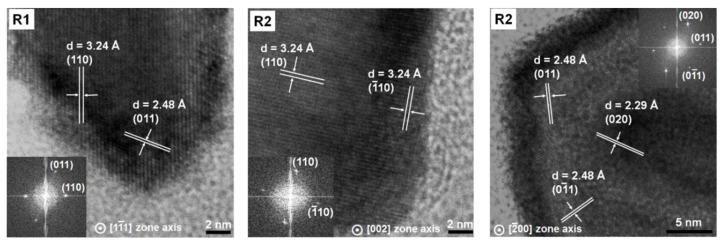
HR-TEM of different rutile samples (R1, R2, and R3 represent the rutile synthesized by the hydrothermal method, rutile separated from the brookite/rutile mixture by peptization with water, and the rutile obtained by the short-time grinding of R1, respectively). Inset: corresponding FFT patterns.

Usually, rutile TiO_2_ crystals show a rod morphology with the competitive growth of {111} facets and {110} facets [38]. It has also been established that the {110} facets include some point defect types, typically bridging oxygen vacancies, which are strongly related to the surface reactivity. Additionally, these facets have a tendency to trap holes and electrons, which greatly yields the improvement in the separation efficiency of electron-hole pairs, and thus the improvement in activity [39,40,41,42,43]. In our case, we found that the rutile TiO_2_ rods with exposed crystal facets that have been predominantly ascribed to the {110} side and {111} edge (sample R1) and {011} edge (sample R2) (Figure 7) had much higher photocatalytic activity than those with a mixture of exposed facets (sample R3 exposing {011}, {020}, and {0$\bar{1}$1} facets). Samples R1 and R2 were both hydrothermal products under different conditions given as 200 °C, HCl 4 M and 175 °C, HCl 3 M, respectively. Under a similar synthesis procedure, these rutile samples were both formed in the rod-shaped nanocrystals with the main active facets of {110}. In contrast, the grinding of R1 may have destroyed or decreased the number of this facet leading to its absence in the R3 sample, which was less active than samples R1 and R2. This finding confirmed the important role of the {110} facets to explain the high photocatalytic activity of the rutile nanorods for the photodegradation of recalcitrant ibuprofen even with a large particle size and low surface area.

## 4. Conclusions

In conclusion, highly crystalline rutile TiO_2_ nanorods were obtained by hydrothermal synthesis at 200 °C using hydrochloric acid as an acidic agent. Rutile had an unexpectedly high photocatalytic activity in the photodegradation of reactive cinnamic acid even with a large crystal size and low specific surface area. The activity of rutile was correlated to the dominant {110} facets in the photodegradation of recalcitrant ibuprofen. Larger proportion of surface exposed {110} active facets has been formed, therefore, higher photocatalytic activity has been achieved. Rutile behaves according to the chemical reactivity of the organic compounds. The lack of oxidative holes caused a lower IBP aromatic ring opening efficiency, whereas the less strong ^•^OH radicals preferentially formed and easily attacked the cinnamic acid molecules.

## Figures and Tables

**Table 1:** Densities of the adsorbed water and surface hydroxyl groups calculated based on the weight loss and S_BET_ for anatase, rutile, and titania P25.

Sample	Weight Loss (%)		S_BET_^1)^ (m^2^/g)	Density of Adsorbed Water (Molecules/nm^2^)	Density of Surface OH Groups (Molecules/nm^2^)
	RT–250 °C	250–700 °C			
Rutile	1.01	0.67	12	28	25
Titania P25	2.65	0.79	46	19	8
Anatase ^2)^	4.86	3.03	132	12	10

^1),2)^ published in Ref. [26].

**Table 2:** Photocatalytic abatement of CA (%) over different rutile samples.

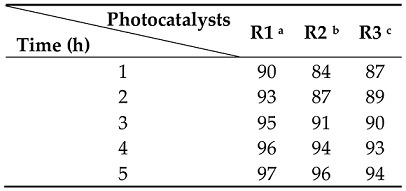

^a^ Rutile synthesized by the hydrothermal method. ^b^ Rutile separated from the brookite/rutile mixture by peptization with water. ^c^ Rutile obtained by the short-time grinding of R1.

## References

[1] Ibhadon A., Fitzpatrick P. (2013). Heterogeneous Photocatalysis: Recent Advances and Applications. Catalysts.

[2] Litter M. (1999). Heterogeneous photocatalysis Transition metal ions in photocatalytic systems. Appl. Catal. B Environ..

[3] Kanakaraju D., Glass B.D., Oelgemöller M. (2014). Titanium dioxide photocatalysis for pharmaceutical wastewater treatment. Environ. Chem. Lett..

[4] Hashimoto K., Irie H., Fujishima A. (2005). Photocatalysis: A Historical Overview and Future Prospects. Jpn. J. Appl. Phys..

[5] Ribeiro A.R., Nunes O.C., Pereira M.F.R., Silva A.M.T. (2015). An overview on the advanced oxidation processes applied for the treatment of water pollutants defined in the recently launched Directive 2013/39/EU. Environ. Int..

[6] Hoffmann M.R., Martin S.T., Choi W., Bahnemann D.W. (1995). Environmental Applications of Semiconductor Photocatalysis. Chem. Rev..

[7] Herrmann J. (1999). Heterogeneous photocatalysis: Fundamentals and applications to the removal of various types of aqueous pollutants. Catal. Today.

[8] Xu H., Ouyang S., Liu L., Reunchan P., Umezawa N., Ye J. (2014). Recent advances in TiO_2_-based photocatalysis. J. Mater. Chem. A.

[9] Tseng T.K., Lin Y.S., Chen Y.J., Chu H. (2010). A review of photocatalysts prepared by sol-gel method for VOCs removal. Int. J. Mol. Sci..

[10] Hanaor D.A.H., Sorrell C.C. (2011). Review of the anatase to rutile phase transformation. J. Mater. Sci..

[11] Kaplan R., Erjavec B., Pintar A. (2015). Enhanced photocatalytic activity of single-phase, nanocomposite and physically mixed TiO_2_ polymorphs. Appl. Catal. A.

[12] Li Z., Cong S., Xu Y. (2014). Brookite vs. Anatase TiO_2_ in the Photocatalytic Activity for Organic Degradation in Water. ACS Catal..

[13] Zhang J., Zhou P., Liu J., Yu J. (2014). New understanding of the difference of photocatalytic activity among anatase, rutile and brookite TiO_2_. Phys. Chem. Chem. Phys..

[14] Kaplan R., Erjavec B., Dražić G., Grdadolnik J., Pintar A. (2016). Simple synthesis of anatase/rutile/brookite TiO_2_ nanocomposite with superior mineralization potential for photocatalytic degradation of water pollutants. Appl. Catal. B.

[15] Jayanthi Kalaivani G., Suja S.K. (2016). TiO_2_ (rutile) embedded inulin—A versatile bio-nanocomposite for photocatalytic degradation of methylene blue. Carbohydr. Polym..

[16] Nair R.V., Jijith M., Gummaluri V.S., Vijayan C. (2016). A novel and efficient surfactant-free synthesis of Rutile TiO_2_ microflowers with enhanced photocatalytic activity. Opt. Mater.

[17] Truong Q.D., Kato H., Kobayashi M., Kakihana M. (2015). Hierarchical structures of rutile exposing high-index facets. J. Cryst. Growth.

[18] Zhang Q., Li R., Li Z., Li A., Wang S., Liang Z., Liao S., Li C. (2016). The dependence of photocatalytic activity on the selective and nonselective deposition of noble metal cocatalysts on the facets of rutile TiO_2_. J. Catal..

[19] Zhang J., Liu P., Lu Z., Xu G., Wang X., Qian L., Wang H., Zhang E., Xi J., Ji Z. (2015). One-step synthesis of rutile nano-TiO_2_ with exposed {111} facets for high photocatalytic activity. J. Alloys Compd..

[20] Reyes-Coronado D., Rodríguez-Gattorno G., Espinosa-Pesqueira M.E., Cab C., de Coss R., Oskam G. (2008). Phase-pure TiO_2_ nanoparticles: Anatase, brookite and rutile. Nanotechnology.

[21] Wei X., Zhu G., Fang J., Chen J. (2013). Synthesis, Characterization, and Photocatalysis of Well-Dispersible Phase-Pure Anatase TiO_2_ Nanoparticles. Int. J. Photoenergy.

[22] Mahshid S., Askari M., Ghamsari M.S. (2007). Synthesis of TiO_2_ nanoparticles by hydrolysis and peptization of titanium isopropoxide solution. J. Mater. Process. Technol..

[23] Tompsett G.A., Bowmaker G.A., Cooney R.P., Metson J.B., Rodgers K.A., Seakins J.M. (1995). The Raman spectrum of brookite, TiO_2_ (*Pbca*, *Z* = 8). J. Raman Spectrosc..

[24] Yang J., Mei S., Ferreira J.M.F., Norby P., Quaresmâ S. (2005). Fabrication of rutile rod-like particle by hydrothermal method: An insight into HNO_3_ peptization. J. Colloid Interface Sci..

[25] Kakuma Y., Nosaka A.Y., Nosaka Y. (2015). Difference in TiO_2_ photocatalytic mechanism between rutile and anatase studied by the detection of active oxygen and surface species in water. Phys. Chem. Chem. Phys..

[26] Tran H.T.T., Kosslick H., Ibad M.F., Fischer C., Bentrup U., Vuong T.H., Nguyen L.Q., Schulz A. (2017). Photocatalytic Performance of Highly Active Brookite in the Degradation of Hazardous Organic Compounds Compared to Anatase and Rutile. Appl. Catal. B.

[27] Bouras P., Stathatos E., Lianos P. (2007). Pure versus metal-ion-doped nanocrystalline titania for photocatalysis. Appl. Catal. B.

[28] Choina J., Bagabas A., Fischer C., Flechsig G.-U., Kosslick H., Alshammari A., Schulz A. (2015). The influence of the textural properties of ZnO nanoparticles on adsorption and photocatalytic remediation of water from pharmaceuticals. Catal. Today.

[29] Carballa M., Omil F., Alder A.C., Lema J.M. (2006). Comparison between the conventional anaerobic digestion of sewage sludge and its combination with a chemical or thermal pre-treatment concerning the removal of pharmaceuticals and personal care products. Water Sci. Technol..

[30] Murakami F.S., Bernardi L.S., Pereira R.N., Valente B.R. (2009). Comparative behavior studies of cinnamic acid using isothermal and nonisothermal kinetic methods. Pharm. Chem. J..

[31] Marques R.R.N., Sampaio M.J., Carrapiço P.M., Silva C.G., Morales-Torres S., Dražić G., Faria J.L., Silva A.M.T. (2013). Photocatalytic degradation of caffeine: Developing solutions for emerging pollutants. Catal. Today.

[32] Wang Y., Shi R., Lin J., Zhu Y. (2010). Significant photocatalytic enhancement in methylene blue degradation of TiO_2_ photocatalysts via graphene-like carbon in situ hybridization. Appl. Catal. B Environ..

[33] Liu J., Liu R., Li H., Kong W., Huang H., Liu Y., Kang Z. (2014). Au nanoparticles in carbon nanotubes with high photocatalytic activity for hydrocarbon selective oxidation. Dalton Trans..

[34] Yang M.-Q., Zhang Y., Zhang N., Tang Z.-R., Xu Y.-J. (2013). Visible-light-driven oxidation of primary C–H bonds over CdS with dual co-catalysts graphene and TiO_2_. Sci. Rep..

[35] Zangeneh H., Zinatizadeh A.A.L., Habibi M., Akia M., Hasnain Isa M. (2015). Photocatalytic oxidation of organic dyes and pollutants in wastewater using different modified titanium dioxides: A comparative review. J. Ind. Eng. Chem..

[36] Murai M., Tamaki Y., Furube A., Hara K., Katoh R. (2007). Reaction of holes in nanocrystalline TiO_2_ films evaluated by highly sensitive transient absorption spectroscopy. Catal. Today.

[37] Beydoun D., Amal R., Low G., McEvoy S. (1999). Role of Nanoparticles in Photocatalysis. J. Nanopart. Res..

[38] Matsunaga K., Tanaka Y., Toyoura K., Nakamura A., Ikuhara Y., Shibata N. (2014). Existence of basal oxygen vacancies on the rutile TiO_2_(110) surface. Phys. Rev. B.

[39] Nakamura R., Okamura T., Ohashi N., Imanishi A., Nakato Y. (2005). Molecular mechanisms of photoinduced oxygen evolution, PL emission, and surface roughening at atomically smooth (110) and (100) *n*-TiO_2_ (rutile) surfaces in aqueous acidic solutions. J. Am. Chem. Soc..

[40] Wallace S.K., Mckenna K.P. (2015). Facet-Dependent Electron Trapping in TiO_2_ Nanocrystals. J. Phys. Chem. C.

[41] Kowalski P.M., Camellone M.F., Nair N.N., Meyer B., Marx D. (2010). Charge localization dynamics induced by oxygen vacancies on the TiO₂(110) surface. Phys. Rev. Lett..

[42] Zuo F., Bozhilov K., Dillon R.J., Le Wang An, Smith P., Zhao X., Bardeen C., Feng P. (2012). Active facets on titanium(III)-doped TiO_2_: An effective strategy to improve the visible-light photocatalytic activity. Angew. Chem. Int. Ed..

[43] Ohno T., Murakami N., Liu R.S. (2012). Murakami. Spatial Separation of Reaction Sites on Rutile TiO_2_ Nanorod. Controlled Nanofabrication: Advances and Applications.

